# Do IGF-I concentrations better reflect growth hormone (GH) action in children with short stature than the results of GH stimulating tests? Evidence from the simultaneous assessment of thyroid function

**DOI:** 10.1186/1756-6614-4-6

**Published:** 2011-01-13

**Authors:** Joanna Smyczyńska, Renata Stawerska, Andrzej Lewiński, Maciej Hilczer

**Affiliations:** 1 Department of Pediatric Endocrinology, Medical University of Lodz, Lodz, Poland; 2 Department of Endocrinology and Metabolic Diseases, Medical University of Lodz, Lodz, Poland; 3 Department of Endocrinology and Metabolic Diseases, Polish Mother's Memorial Hospital - Research Institute in Lodz, Poland

## Abstract

**Background:**

The diagnosis of growth hormone (GH) deficiency (GHD) in short children seems unquestionable when both GH peak in stimulating tests (GHST) and IGF-I concentration are decreased. However, the discrepancies between the results of GHST and IGF-I secretion are observed. It seems purposeful to determine the significance of GHST and IGF-I assessment in diagnosing GHD. The relationship between GH secretion and thyroid function, as well as GH influence on the peripheral thyroxine (T_4_) to triiodothyronine (T_3_) deiodination, mediated by IGF-I, were identified. Thus, clear differences in thyroid function between GH-deficient and non-GH-deficient subjects should exist.

**Methods:**

Analysis comprised 800 children (541 boys), age 11.6 ± 3.1 years (mean ± SD), with short stature, in whom two (2) standard GHST (with clonidine and with glucagon) were performed and IGF-I, free T_4 _(FT_4_), free T_3 _(FT_3_) and TSH serum concentrations were assessed. The patients were qualified to the following groups: GHD - decreased GH peak in GHST and IGF-I SDS (n = 81), ISS - normal GH peak and IGF-I SDS (n = 347), low GH - normal IGF-I SDS, and decreased GH peak (n = 212), low IGF - decreased IGF-I SDS, and normal GH peak (n = 160). The relationships among the results of particular tests were evaluated.

**Results:**

In the groups with decreased IGF-I concentrations (GHD Group and low IGF Group), the more severe deficit of height was observed, together with higher TSH and FT_4 _but lower FT_3 _levels than in groups with normal IGF-I concentrations (ISS Group and low GH Group), independently of the results of GHST. TSH, FT_4 _and FT_3 _concentrations were - respectively - similar in two groups with decreased IGF-I secretion, as well as in two groups with normal IGF-I levels. Significant correlations were found between patients' height SDS and IGF-I SDS, between FT_3 _and IGF-I SDS (positive), and between FT_4 _and IGF-I SDS (negative), with no correlation between GH peak and any of the parameters analyzed.

**Conclusion:**

The assessment of thyroid function in children with short stature provides the evidence that measurement of IGF-I concentration may be a procedure reliable at least to the some degree in diagnosing GHD as the results of GHST.

## Background

The growth hormone (GH) secretion is routinely evaluated in children with short stature, on the ground of the results of GH stimulating tests (GHST) with different pharmacological stimuli [1,2]. Insulin-like growth factor-I (IGF-I) is the main peripheral mediator of GH activity. The assessment of IGF-I secretion is an important diagnostic tool, as its synthesis is GH-dependent, whereas serum concentration - on the contrary to GH levels - relatively stable [3]. The diagnosis of GH deficiency (GHD) seems unquestionable when both GH peak in GHST and IGF-I concentration are decreased. Consistently, GHD may be excluded in the patients, in whom both GH and IGF-I secretion are normal. In such cases, the diagnosis of idiopathic short stature (ISS) seems to be the most adequate after careful exclusion of other possible causes of short stature (including genetically-determined syndromes, chronic diseases, malnutrition, *etc*.). However, in clinical practice, the discrepancies either between the results of GHST and IGF-I secretion or between the results of particular GHST are - quite frequently - observed. According to current recommendations, two (2) different GHST are required in every case and their results should be interpreted together [1,2]. The problem of the poor reproducibility of GHST was raised - among others - by Rosenfeld et al. [4] and Price et al. [5]. Next, the possibility of obtaining normal results of GHST in children, previously diagnosed as GH-deficient (*i.e*. in children with subnormal GH response to pharmacological stimulation in past) was documented in several studies [5-8]. The relationships between GH secretion, more presently - between the results of GHST and IGF-I levels were assessed in numerous studies. Unfortunately, their results are incoherent, either confirming [9-12] or denying [13-16] the correlation between GH peak in GHST and IGF-I secretion. In 2005, Cianfarani et al. [3] stated that IGF-I concentrations were reliable indicators of daily GH secretion, due to their GH dependency and relative stability in circulation. Similar were the results of much earlier observations of Rosenfeld et al. [17] and Shalet et al. [18]. In one of our previous studies [19], the stability of IGF-I concentration was also proved, despite divergent results of the repeated GHST.

Taking into account all the doubts concerning diagnosing GHD in children, it seems purposeful to search for other indices that could be helpful in the evaluation of GH secretory status. Particularly, further studies on determining the significance of both GHST and IGF-I assessment among the diagnostic procedures seem promising.

The relationships between GH secretion and thyroid function, as well as GH influence on the peripheral thyroxine (T_4_) to triiodothyronine (T_3_) deiodination were identified during the observations of patients with GHD, either untreated or subjected to GH therapy. The first studies, carried out about 30 years ago, revealed an increase of T_4 _to T_3 _conversion, leading to an increase of extrathyroidal T_3 _concentration, together with a decrease of T_4 _concentration during GH administration [20,21]. These observations were also confirmed by the results of subsequent studies. In 1989, Jørgensen et al. [22] confirmed the observation that GH replacement induced an enhancement of T_4 _to T_3 _deiodination. In 1994, the same research group [23] demonstrated that in the adult patients with GHD the levels of T_3 _remained decreased, even in the course of T_4 _substitution, while normalizing during GH replacement therapy (parallel to the decrease of TSH levels). The key role of IGF-I in stimulating the process of T_4 _to T_3 _deiodination was also suggested by Jørgensen et al. [24].

On the other hand, the influence of thyroid function disorders on GH secretion, as well as on IGF system, was also described. Thus, it was shown that hypothyroidism (HypoT) - even in its subclinical form - might affect IGF-I secretion [25]. Moreover, it was previously supposed that the effects of thyroid hormone on IGF-I secretion could be independent from GH mediation [26,27]. In particular, a significant decrease of IGF-I and IGF binding protein-3 (IGFBP-3) concentrations was documented in the patients with HypoT, both in adults [28] and in children [29]. Furthermore, it was shown that T_4 _replacement therapy improved the previously decreased GH and IGF-I secretion, however, with no complete recovery [30].

As it was mentioned before, a decrease of T_4 _to T_3 _deiodination, leading to the decrease of T_3 _concentration with reference to T_4 _level, should be observed in GH-deficient patients. Ceratinly, the individual variances of the thyroid function or/and in the activity of peripheral deiodinases must also be taken into account. Nevertheless, it seems that in the study including large groups of patients, the clear differences between GH-deficient and non-GH-deficient subjects should be identified.

The aim of current study was to compare the selected indices of thyroid function in children with short stature, diagnosed towards GHD, with reference to both the results of GHST and IGF-I secretion. In fact, we expected to reveal the differences between the groups with either normal or subnormal results of GHST and/or between those with either normal or decreased IGF-I levels, as since such observations could contribute to optimizing the assessment of GH secretory status in children with short stature.

## Methods

The retrospective analysis comprised 800 children (541 boys, 259 girls), age 11.6 ± 3.1 years (mean ± SD), with short stature (*i.e*., patient's height below 3^rd ^centile for age and sex), diagnosed at our Department (2001-2010). All the children underwent the following diagnostic procedures:

- patients' height was measured on admission to the hospital and was expressed as height SDS (hSDS) for age and sex for Polish children [31]; taking into account the normal distribution of heights in the population of children, an exact value -1.88 was assumed as hSDS for 3^rd ^centile;

- two (2) GHST were performed in children admitted to the hospital, remaining fasting, in the morning hours. For stimulation, clonidine in a dose of 0.15 mg/m^2 ^*p.o*. (1^st ^test), and glucagon in a dose of 30 μg/kg *i.m*., not exceeding 1 mg (2^nd ^test) were used. Blood samples for GH estimation were collected every 30 min from 0 to 120 min in the tests with clonidine and at 0, 90, 120, 150 and 180 min in the test with glucagon;

- serum IGF-I, free T_4 _(FT_4_), free T_3 _(FT_3_) and TSH concentrations were measured in single blood samples, obtained in 0 min of 1^st ^stimulating test, just before clonidine administration.

The main assumption of the study was to include the patients either with ISS or with isolated non-acquired GHD only. In order to avoid the possible influence of disorders of thyroid function on GH and/or IGF-I secretion and action, only the children with values of TSH, FT_4_, and FT_3 _concentrations within the reference range were included into the analysis. The exclusion criteria encompassed any chronic diseases, other hormonal deficiencies, acquired GHD, Turner syndrome in girls, other genetically determined syndromes, malnutrition and obesity (assessed as body mass index either below or over the reference range respectively for age and sex [31]).

The concentrations of GH were measured by the two-site chemiluminescent enzyme immunometric assay (hGH IMMULITE, DPC) for the quantitative measurement of human GH, calibrated to WHO IRP 80/505 standard. The analytical sensitivity of the assay was up to 0.01 ng/ml, the calibration range up to 40 ng/ml, the sensitivity of 0.01 ng/ml, the intra-assay coefficient of variation (CV) of 5.3-6.5% and the inter-assay CV of 5.5-6.2%. The cut-off value for normal and subnormal GH peak in response to stimulation was 10.0 ng/ml according to current recommendations [1,2].

Serum IGF-I concentrations were assessed by Immulite, DPC assays; WHO NIBSC 1^st ^IRR 87/518 standard was applied, with analytical sensitivity 20 ng/ml, calibration range up to 1600 ng/ml, intra-assay CV - 3.1-4.3% and inter-assay CV - 5.8-8.4%. For comparison among children of different age and sex, IGF-I concentrations were expressed as IGF-I SDS, according to DPC reference data. As it was stated before, IGF-I values higher than -1.0 SD reflected a normal GH secretion [3]. Thus, we decided to select just that threshold value as the cut-off level for normal and decreased IGF-I secretion in our study.

Serum TSH, FT_4 _and FT_3 _concentrations were measured by the electroimmunochemiluminescent method (ECLIA), Roche, Elecsys^®^Systems 1010/2010/modular analytics E170. For TSH, the analytical sensitivity was 0.005 μIU/ml, range - up to 100 μIU/ml, intra-assay coefficient of variance (CV) - 1.5-8.6%, accuracy - 1.1-3.0%. The analytical range for FT_4 _was 0.023-7.77 ng/ml, intra-assay CV - 1.4-2.9%, accuracy - 2.7-6.6%. For FT_3_, the analytical range was 0.26-32.55 pg/ml, intra-assay CV - 3.7-9.5%, accuracy - 3.8-11.2%. For the assessment of FT_3_/FT_4 _molar ratio, the concentrations of FT_4 _and FT_3 _were expressed as molar ones.

According to the results of GHST (normal or subnormal) and to IGF-I secretion (normal or decreased), the patients were qualified to the following groups:

- **GHD **- the patients with decreased both GH peak in GHST and IGF-I SDS (n = 81);

- **ISS **- the patients with normal both the results of GHST and IGF-I SDS (n = 347);

- **low GH **- the patients with normal IGF-I SDS, despite decreased GH peak in GHST (n = 212);

- **low IGF **- the patients with decreased IGF-I SDS, despite normal GH peak in GHST (n = 160).

The analysis of the relationships between the components of somatotrophic axis (the results of GHST, IGF-I concentration) and the components of the pituitary - thyroid axis (TSH, FT_4 _and FT_3 _concentrations) were assessed. Statistical analysis included Kruskal-Wallis' nonparametric test for independent samples, as well as the assessment of correlations between patient's height and the results of particular hormonal tests.

## Results

The deficit of height (expressed as hSDS for age and sex) proved to be the most expressed in **low IGF Group **(*i.e*. in the patients with low IGF-I and normal GH peak in GHST), being significantly lower than that in both **ISS Group **(p < 0.001) and **low GH Group **(p < 0.001), as well as than that in **GHD Group **(p = 0.031). Interestingly, both the mean value and the distribution of hSDS values in **ISS Group **and in **low GH Group **(*i.e*. in the groups of patients with normal IGF-I secretion) were similar, despite completely different results of GHST in them. Moreover, hSDS values in these groups were also higher than those in the groups with decreased IGF-I, *i.e*. **GHD Group **and **low IGF Group **(also independently of the results of GHST).

The levels of TSH were also very similar in **ISS Group **and in **low GH Group**, being - at the same time - lower than both in **GHD Group **(the differences insignificant - NS) and than in **low IGF Group **(NS and p = 0.032, respectively), while the difference between **GHD Group **and **low IGF Group **was insignificant.

Moreover, FT_4 _concentrations turned out to be very similar in **ISS Group **and in **low GH Group**, being also significantly lower than both in **GHD Group **(p < 0.001 and p = 0.002, respectively) and in **low IGF Group **(p < 0.001 for both pairs compared).

Interestingly, there was no significant difference in FT_3 _concentrations between **ISS Group **and **low GH Group**, while in these two groups, FT_3 _concentrations were significantly higher than those in both **GHD Group **(p = 0.012 and p = 0.010, respectively) and **low IGF Group **(p = 0.001 for both pairs compared). The difference between **GHD Group **and **low IGF Group **did not reach the border of significance.

**Figure 1:**
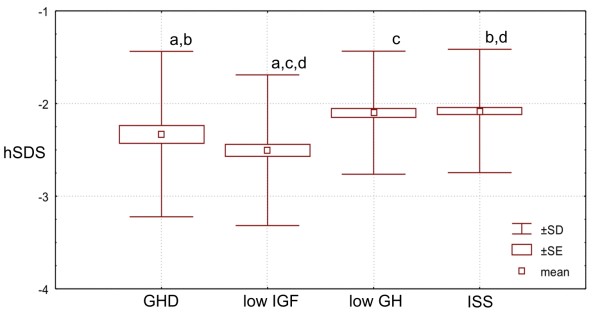
**Patients' height (expressed as hSDS) in particular groups of children**. Significant differences: a,b - p < 0.05; c,d - p < 0.001.

**Figure 2:**
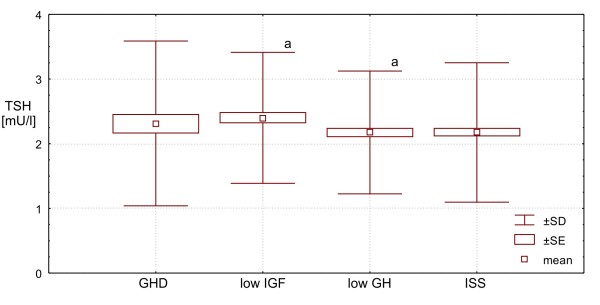
**TSH secretion in particular groups of patients. Significant difference: a - p < 0.05**.

**Figure 3:**
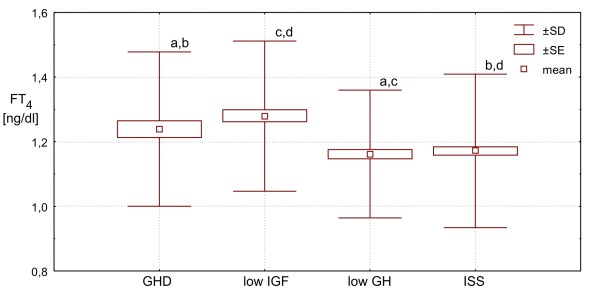
**FT**_**4 **_**secretion in particular groups of patients. Significant differences: a - p < 0.005; b,c,d - p < 0.01**.

**Figure 4:**
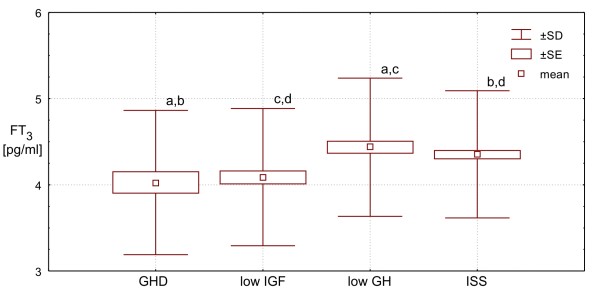
**FT**_**3 **_**concentrations in particular groups of patients. Significant differences: a - p < 0.01; b,c,d - p < 0.001**.

**Figure 5:**
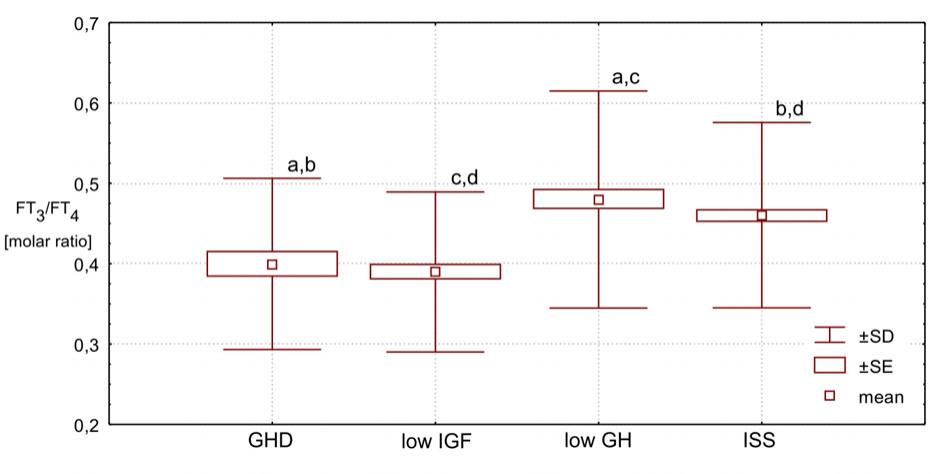
**Molar ratio FT**_**3**_**/FT**_**4 **_**in particular groups of patients. Significant differences: a,b,c,d - p < 0.0001**.

Finally, FT_3_/FT_4 _molar ratio was similar in **ISS Group **and **low GH Group**, being - in these two groups - significantly higher (p < 0.0001) than those in **GHD Group **and in **low IGF Group**, while the difference between the latter two groups was also insignificant. More detailed data, are presented Table 1 and Figures 1, 2, 3, 4 and 5.

**Table 1:** Patients' height and thyroid function in particular groups of children.

Group	GHD	low IGF-I	low GH	ISS
**max GH **[ng/ml] **IGF-I SDS**	< 10 ng/ml < -1.0	≥10 ng/ml < -1.0	< 10 ng/ml ≥-1.0	≥10 ng/ml ≥-1.0

**hSDS **for age and sex	-2.31 ± 0.88 ^a,b^	-2.50 ± 0.81 ^a,c,d^	-2.10 ± 0.66 ^c^	-2.08 ± 0.67 ^b,d^

**TSH **[mIU/l]	2.33 ± 1.27	2.40 ± 1.01 ^e^	2.17 ± 0.75 ^e^	2.17 ± 1.08

**FT**_4 _[ng/dl]	1.25 ± 0.23 ^f,g^	1.28 ± 0.23 ^h,i^	1.16 ± 0.20 ^f,h^	1.17 ± 0.24 ^g,i^

**FT**_3 _[pg/ml]	4.03 ± 0.83 ^j,k^	4.09 ± 0.80 ^l,m^	4.44 ± 0.80 ^j,l^	4.35 ± 0.74 ^k,m^

**FT**_3_/**FT**_4 _[molar ratio]	0.40 ± 0.11 ^n,o^	0.39 ± 0.10 ^p,r^	0.48 ± 0.14 ^n,p^	0.46 ± 0.12 ^o,r^

Significant differences: a,b - p < 0.05; c,d - p < 0.001; e-p < 0.05; f-p < 0.005; g,h,i,j - p < 0.01; k,l,m - p < 0.001; n,o,p,r - p < 0.0001

Summing up, in groups of patients with decreased IGF-I concentrations (*i.e*. in **GHD Group **and in **low IGF Group**), the more severe deficit of height was observed, together with higher TSH and FT_4 _levels but lower FT_3 _concentrations than those in groups with normal IGF-I concentrations (*i.e*. in **ISS Group **and in **low GH Group**). All the assessed parameters of thyroid function presented similar in the two groups with decreased IGF-I secretion (*i.e*. **GHD Group **and **low IGF Group**). There were no important differences between groups with normal IGF-I levels (*i.e*. **ISS Group **and **low GH Group**).

As a matter of fact, there were no strong correlations between the assessed indices of thyroid function (TSH, free thyroid hormones) and the parameters of somatotrophic axis (GH peak in GHST, IGF-I). However, a significant, positive correlation was found between patients' hSDS and IGF-I SDS (r = 0.25, p < 0.05), while there was no correlation between hSDS and GH peak in GHST (r = 0.03, NS). A positive correlation was also observed between IGF-I SDS and FT_3 _concentration (r = 0.24, p < 0.05), together with a negative one between IGF-I SDS and FT_4 _(r = -0.20, p < 0.05) and with no correlation between IGF-I SDS and TSH level (r = -0.06, NS). Thus, the observed correlations seem to be independent from TSH level, while an increase of IGF-I concentration may be related to the increase of FT_3 _and decrease of FT_4_. Moreover, there was no correlation between GH peak and any of the parameters analyzed. Detailed data are shown in Table 2.

**Table 2:** Correlations between the indices of thyroid function and the components of somatotrophic axis.

	hSDS	GH peak [ng/ml]	IGF-I SDS	TSH [mIU/l]	**FT**_4 _ [ng/dl]	**FT**_3 _ [pg/ml]
**hSDS**		0.03	0.25*	-0.05	0.05	0.13*

**GH peak **[ng/ml]	0.03		0.12*	0.01	0.12*	0.00

**IGF-ISDS**	0.25*	0.12*		-0.06	-0.20*	0.24*

**TSH **[mIU/l]	-0.05	0.01	-0.06		0.12*	0.10*

**FT**_4 _[ng/dl]	0.05	0.12*	-0.20*	0.12*		0.03

**FT**_3 _[pg/ml]	0.13*	0.00	0.24*	0.10*	0.03	

Correlations marked * are significant (p < 0.05)

## Discussion

In our study, groups with decreased IGF-I levels (*i.e*., GHD Group and low IGF-I Group) presented with the relatively higher TSH and FT_4 _but lower FT_3 _concentrations than those with normal IGF-I secretion, independently from either subnormal or normal GH peak in GHST. It should be recalled that GHD leads to the decrease of peripheral T_4 _to T_3 _deiodination [20-23]. Thus, the observed lower FT_3 _concentrations in both groups with decreased IGF-I, despite even higher FT_4 _levels in them seem to correspond with GHD in these patients. Moreover, the very similar FT_4_, FT_3 _and TSH concentrations in both groups with normal IGF-I levels (*i.e*., in ISS Group and low GH Group), independently from either normal or subnormal results of GHST, should be stressed. We are convinced that these observations speak against the disorders of thyroid function related to GHD in both groups with normal IGF-I secretion, especially since we certainly do not expect such disorders in patients with ISS.

According to previous suggestions that IGF-I may be a mediator of GH action on stimulating peripheral T_4 _deiodination [24], the results of current study seem quite reliable. The quoted observation allows to explain the results obtained in GHD Group, ISS Group, and - to some extent - in low IGF Group. In fact, in the latter group, either decreased GH sensitivity or other completely overlooked diseases, present in most of children, should be assumed as the only possible cause of decreased IGF-I secretion. Taking into account both the relatively low incidence of GH insensitivity and the exclusion criteria, such possibility seems, however, poorly justified. Next, the results, obtained in low GH Group do not seem to be explained by anything - except for the falsely positive (*i.e*., falsely decreased) GH peaks in both performed GHST. In these patients, not only IGF-I concentrations are normal but also they correspond to the similar thyroid status as we observed in ISS Group.

As it was mentioned before, the discrepancies between the results of GHST and IGF-I secretion were reported in numerous studies [9-12], being explained either by the individual differences in GH sensitivity [32] or by the lack of concordance between GH secretion under physiological conditions and the results of GHST [33-35]. The conclusions, derived from these observations were also non-consistent. For instance, Rasat et al. [36] proposed IGF-I assessment as a screening procedure in diagnosing GHD. Similar was the statement of Rosenfeld [37-39]. However, there is also a strong evidence that it may be impossible to predict the results of GHST on the ground of IGF-I concentration [9,12-14,16,40-42]. It seems very important to adequately answer the question, which (if any) of these procedures is the most reliable one in GHD diagnosing. According to current recommendations [1,2], GHST are the main tools for the assessment of GH secretion. However, some other options were also proposed. Thus, Badaru and Wilson [43] stated that IGF-I assessment had of no less importance than the results of GHST. Similarly, Loche at al. [7] suggested that the diagnosis of GHD should not exclusively be based on the results of GHST. In 2009, Lemaire et al. [44] proposed the assessment of growth rate and IGF-I concentration as the screening procedures in children suspected for GHD, in order to reduce the necessity of subjecting the patients to GHST. The results of our previous studies [19,45] also speak for the significance of IGF-I assessment in GHD diagnosing, thus bringing into question the credibility of GHST.

Another issue, however not analyzed in the current study, is an assessment of spontaneous GH secretion. Although - up to now - this procedure has not been recommended for clinical practice according to international guidelines [2], the data exist that GH administration to short children with decreased stimulated but normal spontaneous GH secretion is not associated with an increase of final height [46]. It seems that not only decreased spontaneous GH secretion should be taken into account in short children with low IGF-I concentrations (leading to the diagnosis of neurosecretory dysfunction) but also normal spontaneous GH secretion must be considered in the patients with normal IGF-I levels, despite decreased GH peak in GHST. It should also be taken into account that the relatively high incidence of falsely positive results of the two (2) GHST, performed in an individual patient was revealed in one of our previous studies [47]. As in Poland the assessment of spontaneous GH secretion after falling asleep was introduced as an obligatory procedure (screening) in diagnosing GHD in children, our research team intends to assess prospectively both IGF-I secretion and thyroid function with respect to nocturnal GH secretion.

## Conclusion

It seems that the assessment of thyroid function, while diagnosing GHD in children with short stature, provides the evidence that IGF-I concentration may be no less reliable indicator of GH action than the results of GHST, at least in children suspected for idiopathic, isolated GHD and after exclusion of other causes of impaired IGF-I secretion.

## Abbreviations

CV: coefficient of variation; FT_4_: free thyroxine; FT_3_: free triiodothyronine; GH: growth hormone; GHD: growth hormone deficiency; GHST: growth hormone stimulating tests; hSDS: height standard deviation score; Hypo-T: hypothyroidism; IGF-I: insulin-like growth factor-I; IGFBP-3: insulin-like growth factors binding protein-3; ISS: idiopathic short stature; SD: standard deviation; SDS: standard deviation score; T_4_: thyroxine; T_3_: triiodotyronine; TSH: thyroid stimulating hormone (thyrotropin)

## Competing interests

The authors declare that they have no competing interests.

## Authors' contributions

JS designed the study, qualified the patients and performed statistical analysis. RS participated in performing hormonal studies and in statistical analysis. AL participated in drawing up the study protocol and coordinated the study. MH, the senior author, wrote the manuscript. All authors read and approved the final manuscript
